# Establishing a 3D In Vitro Hepatic Model Mimicking Physiologically Relevant to In Vivo State

**DOI:** 10.3390/cells10051268

**Published:** 2021-05-20

**Authors:** Hyun Kyoung Kang, Madina Sarsenova, Da-Hyun Kim, Min Soo Kim, Jin Young Lee, Eun-Ah Sung, Myung Geun Kook, Nam Gyo Kim, Soon Won Choi, Vyacheslav Ogay, Kyung-Sun Kang

**Affiliations:** 1 Adult Stem Cell Research Center, College of Veterinary Medicine, Seoul National University, Seoul 08826, Korea; gusrudk100@snu.ac.kr (H.K.K.); dahyun0515@snu.ac.kr (D.-H.K.); iamcellist@snu.ac.kr (M.S.K.); kmg369@snu.ac.kr (M.G.K.); ngkim93@snu.ac.kr (N.G.K.); mszagm2007@snu.ac.kr (S.W.C.); 2 Stem Cell Laboratory, National Center for Biotechnology, 13/5 Qorgalzhin Highway, Nur-Sultan 010000, Kazakhstan; sarsenova@biocenter.kz (M.S.); ogay@biocenter.kz (V.O.); 3 Department of Medicine, Cardiovascular Research Institute, UCSF, San Francisco, CA 94143, USA; twinklejy1@gmail.com; 4 Department of Cell Biology and Molecular Medicine, Rutgers New Jersey Medical School, Newark, NJ 07103, USA; sungeunah92@gmail.com

**Keywords:** 3D bioprinting, dynamic environment, in vitro model, liver

## Abstract

Three-dimensional (3D) bioprinting is a promising technology to establish a 3D in vitro hepatic model that holds great potential in toxicological evaluation. However, in current hepatic models, the central area suffers from hypoxic conditions, resulting in slow and weak metabolism of drugs and toxins. It remains challenging to predict accurate drug effects in current bioprinted hepatic models. Here, we constructed a hexagonal bioprinted hepatic construct and incorporated a spinning condition with continuous media stimuli. Under spinning conditions, HepG2 cells in the bioprinted hepatic construct exhibited enhanced proliferation capacity and functionality compared to those under static conditions. Additionally, the number of spheroids that play a role in boosting drug-induced signals and responses increased in the bioprinted hepatic constructs cultured under spinning conditions. Moreover, HepG2 cells under spinning conditions exhibited intensive TGFβ-induced epithelial-to-mesenchymal transition (EMT) and increased susceptibility to acetaminophen (APAP)-induced hepatotoxicity as well as hepatotoxicity prevention by administration of N-acetylcysteine (NAC). Taken together, the results of our study demonstrate that the spinning condition employed during the generation of bioprinted hepatic constructs enables the recapitulation of liver injury and repair phenomena in particular. This simple but effective culture strategy facilitates bioprinted hepatic constructs to improve in vitro modeling for drug effect evaluation.

## 1. Introduction

Three-dimensional (3D) bioprinting technology is an emerging approach that has provided researchers an opportunity to fabricate personalized and complex tissue constructs with systemic 3D microarchitecture capable of integration into the native microenvironment [1,2]. Additionally, this technology can be used to create in vitro models for studies of disease mechanisms and drug screening and as a prospective tool in regenerative medicine [3,4,5]. Among 3D bioprinting methods, extrusion-based bioprinting is one of the most commonly used techniques that enables the deposition of high concentrations of cells within biocompatible biomaterials to reconstruct engineered tissue in a layer-by-layer manner [2,6]. This approach potentially represents the most appropriate method to fabricate large-scale engineered tissue, such as the liver, if its main limitations are overcome. These limitations include low cell viability caused by pneumatic pressure-induced shear stress and hypoxic conditions in the core of large-scale bioprinted constructs resulting from limited control of cell–cell and cell–matrix interactions that hamper the long-term culture period [7,8].

The liver is the largest organ with complex architecture and mainly consists of hepatocytes, which comprise approximately 80% of the liver mass, and other cell types. Additionally, the liver primarily metabolizes toxins and drugs first absorbed by the gastrointestinal tract before entering the bloodstream. Indeed, the liver has a dual blood supply circulation system that primarily involves a nutrient-rich vein and oxygen-rich artery [9]. Constant and sufficient amounts of oxygen and nutrient supplements enable the high regeneration capacity of the liver, which is one of its important characteristics compared with other organs. Given the liver’s major roles in exogenous xenobiotic metabolism and detoxification, an appropriate in vitro hepatic model is still needed [10,11,12]. In an in vitro hepatic model, it is important to represent the high metabolism of drugs and toxins resulting from the complex liver microenvironment [13]. Despite many attempts to establish in vitro models, these models do not provide a dynamic microenvironment that is relevant to biochemical and biophysical processes in the liver. Nevertheless, even developed biocompatible extracellular matrices generate cell–ECM and cell–cell interactions and are able to maintain cellular properties in current in vitro hepatic models; however, they remain slightly different from native tissue. Thus, another strategy needs to be created to reflect the complete in vivo microenvironment.

Although many studies on the effect of drugs in the liver were previously conducted using animal models, many candidate drugs failed to demonstrate efficacy in humans due to species differences [14,15]. The situation is exacerbated by increasing ethical issues over animal-based tests, resulting in restricted animal experiments worldwide [16,17,18]. These limitations accelerated the development of better predictive platforms that can complement the existing approaches for drug discovery and testing [16]. In this regard, refined 3D culture methods were discovered to overcome the mentioned restrictions [14,17,19,20,21].

Here, we constructed hexagonal bioprinted constructs using commercial laminin-521-enriched bioink to provide an environment for cells that progressively increased viability and maintained intrinsic functions. Additionally, continuous spinning conditions generated by orbital shakers improved the growth and functionality of encapsulated cells within bioprinted constructs over 2 weeks compared to static conditions. We observed obvious TGF-β-induced epithelial-to-mesenchymal transition and restoration induced by treatment with the TGF-β inhibitor SB431542 under spinning conditions but not under static conditions. Furthermore, spinning conditions recapitulated liver injury and repair phenomena, increasing susceptibility to acetaminophen-induced hepatotoxicity and alleviation by administration of N-acetylcysteine (NAC) in bioprinted hepatic constructs. These results indicate that bioprinted constructs generated under spinning conditions could represent a better strategy for establishing a 3D in vitro hepatic model that is physiologically relevant to the in vivo state.

## 2. Materials and Methods

### 2.1. HepG2 Cell Line Culture

Human hepatocarcinoma cells (HepG2 cells) purchased from ATCC was cultured as a monolayer in high-glucose Dulbecco’s Modified Eagle’s Medium (DMEM; SH3002201; HyClone, Logan, UT, USA) supplemented with 10% fetal bovine serum (FBS; 26140079; Gibco, Carlsbad, CA, USA) and 0.2% Primocin (ant-pm-1; InvivoGen, San Diego, CA, USA) at 37 °C with 5% CO_2_. Cells with 80% confluency were subcultured after dissociation with 0.25% trypsin (GIB-25200-072; Invitrogen, Carlsbad, CA, USA). The medium was changed every other day.

### 2.2. Bioprinting of 3D Hepatic Constructs

GelXA LAMININK and Cellink BIO X 3D pneumatic bioprinters (Cellink, Boston, MA, USA) were used to fabricate functional 3D bioprinted hepatic constructs at room temperature. Hexagonal structures (3 × 3 × 2 mm^3^) mimicking three hepatic lobules were designed using the Tinkercad online 3D modeling program (https://www.tinkercad.com, accessed on 1 May 2021). Cells were prepared at a final density of 3 × 10^7^ cells/mL and mixed with commercial bioinks based on gelatin, alginate, xanthan gum and LAMININ a5b2y1 (IK3 × 21270301, GelXA LAMINIK521, Cellink). Bioink mixed with the cells was loaded into syringes fitted with a 0.25-gauge nozzle and printed on an ultralow-attachment 6-well plate (3471, Costar, Washington, DC, USA). Bioprinted structures were crosslinked for 5 min with a crosslinking agent containing 50 mM calcium chloride provided by Cellink Company (CL1010001501, Cellink). The hepatic constructs were cultured in 4 mL of high-glucose DMEM supplemented with 10% FBS at 37 °C in a humidified atmosphere containing 5% CO_2_. For spinning conditions, culture plates were placed in a platform orbital shaker (88881124, Thermo Fisher Scientific, Waltham, MA, USA) at 60 rpm. The medium was changed every other day.

### 2.3. Live/Dead Cell Staining

Live/dead cell staining was conducted on days 1, 3, 5 and 7 after printing to analyze cell viability in 3D printed hepatic constructs. Calcein AM (15560597, Invitrogen) and propidium iodide (PI; 81845-25MG, Sigma-Aldrich, Saint-Louis, MO, USA) were used at the final concentrations of 0.25 μM and 20 μg/mL, respectively. The images were taken by a confocal microscope (Nikon, Shinagawa, Tokyo, Japan).

### 2.4. Quantitative RT-PCR

Total RNA was extracted using TRIzol (BRL-15596-018, Invitrogen) according to the manufacturer’s instructions. cDNA was synthesized from isolated RNA and detected by real-time PCR using SYBR Green PCR Master Mix (4309155, Applied Biosystems, Foster City, CA, USA) and QuantStudio3 (Applied Biosystems). The expression level of each gene was normalized to GAPDH. At least three independent analyses were conducted for each gene. The results were analyzed using Quantstudio Design and Analysis software v1.4.

### 2.5. Histological Characterization (H&E Staining)

The bioprinted hepatic constructs were collected and washed 3 times with phosphate-buffered saline (PBS; SH30256.01, GE Healthcare Life Sciences, Buckinghamshire, UK). The constructs were fixed with 4% paraformaldehyde (PFA; 158127, Sigma-Aldrich) at 4 °C overnight. The fixed bioprinted structures were processed following a typical method, including dehydration with ethanol, clearing with xylene and wax infiltration with paraffin. Paraffin-embedded blocks were sectioned to 5 μm thickness. Sliced sections were deparaffinized using xylene (1330-20-7, Duksan, Ansan, Republic of Korea) and graded alcohols (64-17-5, Duksan). Samples were stained with hematoxylin (HX73999849, Merck, Darmstadt, Germany) for 30 s and eosin (3200-2, Muto Pure Chemicals, Tokyo, Japan) for 5 min. Finally, samples were washed with running water, rehydrated with graded alcohols and mounted with Canada balsam (C1795, Sigma) for further visualization by microscopy as previously described [22].

### 2.6. Immunocytochemistry

Paraffin slides were deparaffinized, rehydrated and heated for antigen retrieval using sodium citrate solution (pH 6.0) at 95 °C for 20 min. The samples were blocked with 5% normal goat serum (K-S-10000-K13, Vector Laboratories, Burlingame, CA, USA) for one hour at room temperature. The slides were then washed thrice with PBS and incubated with primary antibodies using mouse anti-cytokeratin 18 (MAB3234, Millipore, Burlington, WI, USA) and rabbit anti-albumin (102419, GeneTex, Irvine, CA, USA) at a 1:100 dilution at 4 °C overnight. Alexa Fluor mouse 488 (A1100, Invitrogen) and Alexa Fluor rabbit 555 (A-21428, Invitrogen) secondary antibodies were applied at a 1:1000 dilution for 1 h at room temperature. After washing, fluorescent staining was performed with DAPI solution at 1:1000 in PBS for nuclear detection. The sections were mounted with DAKO fluorescence mounting medium (Agilent Pathology Solutions, Santa Clara, CA, USA) and examined by confocal microscopy (Eclipse TE200, Nikon) as previously described [23]. The list of antibodies used for immunostaining is provided in Supplementary Table S1.

### 2.7. Functional Analysis

The amounts of secreted human albumin, alpha-fetoprotein, alpha-1 antitrypsin and urea from collected cell supernatants were measured using Human Albumin ELISA Quantification Kit (E80-129, Bethyl Laboratories, Montgomery, AL, USA), Human Alpha Fetoprotein ELISA Kit (ab108388, Abcam, Cambridge, UK), Human Alpha-1 Antitrypsin ELISA (ab108799, Abcam) and QuantiChrom Urea Assay Kit (DIUR-100, Bioassay system, Hayward, CA, USA) according to the manufacturer’s instructions.

### 2.8. Western Blot Analysis

The protein concentration was measured using a Pierce BCA Protein Assay Kit (23227, Thermo Fisher Scientific). Approximately 10 μg of protein from each cell was subjected to 8% to 15% sodium dodecyl sulfate polyacrylamide gel electrophoresis and then transferred onto a nitrocellulose membrane. The membranes were blocked with 3% bovine serum albumin solution in TBST, followed by 1 h incubation. After that, the membrane was incubated with primary antibody at 4 °C overnight. The primary antibodies used to probe each protein were as follows: mouse anti-phosphoSmad2/3 (8828, Cell Signaling, Danvers, MA, USA): 1:1000, rabbit anti-Smad2/3 (8685, Cell Signaling): 1:1000, anti-fibronectin (ab2413, Abcam): 1:1000, mouse anti-β-actin (4967, Cell Signaling): 1:1000, mouse anti-phospho-histone H2A.X (05-636, Merck): 1:1000, rabbit anti-cleaved caspase3 (9664, Cell Signaling): 1:1000, rabbit anti-caspase3 (9662, Cell Signaling): 1:1000, rabbit anti-p62 (610832, BD Bioscience, Franklin Lakes, NJ, USA): 1:500 and rabbit anti-LC3 (NB100-2331, Novus Biologicals, Centennial, CO, USA): 1:1000. Secondary horseradish peroxidase (HRP)-conjugated antibodies (G21040, G21234, Invitrogen): 1:2000. The antibody binding was detected using an enhanced chemiluminescence (ECL) detection kit (RPN2106, GE Healthcare Life Science).

### 2.9. Drug (Acetaminophen, N-Acetyl-L-cysteine) Treatment

For the drug sensitivity assessment, bioprinted hepatic constructs were cultured under spinning conditions for 14 days. Then, the constructs were treated with N-acetyl-L-cysteine (A7250, Sigma-Aldrich) for 12 h. Then, culture supernatants were collected. On day 14.5, bioprinted hepatic constructs were treated with acetaminophen (APAP; A3036-1VL, Sigma-Aldrich) at a working concentration of 15 mM for 48 h. Supernatants were collected every 48 h for 8 days and frozen at −80 °C for human albumin ELISA and urea production. On the final day, the bioprinted structures were fixed with 4% PFA and embedded in paraffin for histological characterization.

### 2.10. Statistical Analysis

Statistical analyses were performed using GraphPad Prism version 9 Software. In addition, the data are presented as representative examples or mean values when more than three experiments were conducted. Data are presented as the means ± S.D. Two-tailed Student’s *t*-test was performed to compare the data from two groups, or one-way ANOVA followed by Bonferroni’s test was performed to compare data from multiple groups throughout our experiments.

## 3. Results

### 3.1. Fabrication of 3D Bioprinted Human Liver Tissue

**Figure 1:**
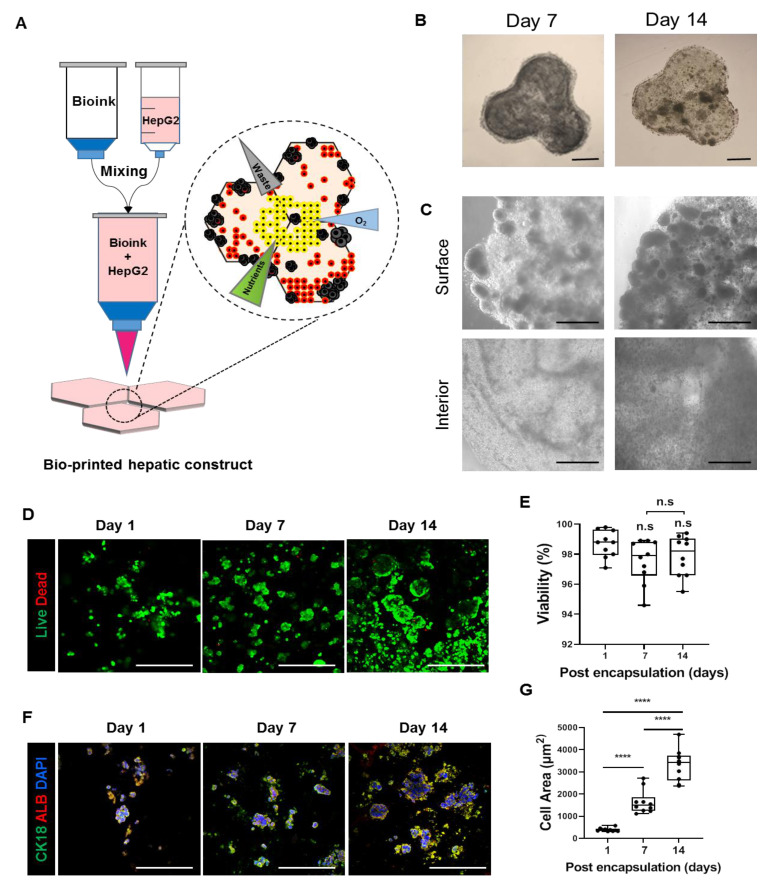
Fabrication of a 3D bioprinted hepatic construct. (**A**) Schematic illustration for fabrication of 3D bioprinted hepatic constructs encapsulated with HepG2 cells. (**B**,**C**) Bright-field images for bioprinted hepatic constructs on days 7 and 14. Cells self-aggregated into spheroid-like structures. (**B**) Whole construct, Scale bar = 1000 μm. (**C**) Surface and interior areas. Surface area of spheroids is greater compared to interior area. Scale bar = 1000 μm. (**D**) Representative live/dead images of encapsulated cells in 3D bioprinted hepatic constructs on days 7 and 14 stained with calcein AM (green) and PI (red). Scale bar = 500 μm. (**E**) Quantification of cell viability from live/dead images. Bar graph represents viability of HepG2 cells in bioprinted hepatic constructs at indicated time points. n.s: no significance. (**F**) Representative immunofluorescence images of HepG2 cells encapsulated in the 3D bioprinted hepatic construct on days 7 and 14. Sections were stained with CK18 (green) and ALB (red). Scale bar = 500 μm. (**G**) Quantification of cell area of CK18 and ALB double-positive stained cells. Bar graph represents calculated cellular area of encapsulated cells that expressed hepatic marker in bioprinted hepatic constructs at indicated time points. All error bars represent the means ± S.D. from three separate experiments. **** *p* < 0.0001.

To create artificial hepatic tissue that mimics native architecture and microenvironment, we encapsulated HepG2 cells, a widely used hepatocarcinoma cell line, in a hexagonal digital pattern with dimensions adjusted to the approximate size of one liver lobule unit. In the first step, three hexagonal units (3 × 3 × 2 mm^3^ each unit) anatomically resembling liver lobules were designed to follow human liver tissues in vivo (Figure 1A). A laminin-enriched crosslinkable biomixture provided by Cellink company was used as a bioink. Based on previous studies, laminin-enriched bioink was selected to support the maintenance of hepatic profile expression and clonal expansion of encapsulated cells that have the ability to migrate within bioprinted structures after bioprinting [24,25]. Initially, encapsulated HepG2 cells were well distributed based on the movement of the bioprinter nozzle. On day 7 after bioprinting, cells started to aggregate by themselves in a spheroid-like fashion and increased in size at the edge of bioprinted hepatic constructs until day 14 (Figure 1B,C). Consequently, a number of the cells started to relocate out of the structure. We further questioned whether cells in isolated spheroids have relevant hepatic phenotypes and functionality. Although separated spheroids showed liver cell characteristics confirmed by H&E, PAS staining and immunostaining, these spheroids had hypoxic core areas resulting from the absence of extracellular matrix (Supplementary Figure S2). Previous data report [26] similar results in observed data which showed necrotic central region with some cells and cell debris (Supplementary Figure S2). Additionally, 7 days following bioprinting, single cells located in the interior area of the bioprinted hepatic construct started to aggregate into small-diameter spheroids 7 days after bioprinting (Figure 1B,C). These results suggest that the surface area of the bioprinted hepatic construct was amenable to the motility of encapsulated cells resulting from sufficient supplementation of nutrients and oxygen to form spheroids compared to the interior. Additionally, these findings implied that a prolonged culture period with the current bioprinted hepatic model will reveal progressive problems and that the method should be improved. To evaluate scaffold biocompatibility, we tested which laminin-enriched bioink developed by Cellink would maintain cell survival, growth and hepatic features of HepG2 cells. On day 7, the highest cell viability and proliferation capacity were observed when printed with GelXA LAMININK521 (Supplementary Figure S1A,C). Quantification of live cell numbers showed that on day 7, cell viability in bioprinted hepatic constructs was the highest in the hepatic construct printed with GelXA LAMININK521 (Supplementary Figure S1B). These results suggest that GelXA LAMININK521, unlike other types of bioink, allowed encapsulated cells to extend the culture period with consistent cell viability. Furthermore, we monitored hepatic constructs bioprinted with GelXA LAMIININK521 over 14 days to determine whether the cell culture period could be extended. Hepatic constructs bioprinted with GelXA LAMININK521 maintained cell viability (Figure 1D,E) and constant hepatic marker protein expression for 14 days (Figure 1F). In addition, we tested whether the bioink can improve the hepatic function of HepG2 cells in terms of gene expression. *PROX1*, *CYP1A2* and *CYP3A4* mRNA expression showed significant differences in bioprinted hepatic constructs mixed with GelXA LAMININK521 compared to other groups at 14 days following bioprinting (Supplementary Figure 1D). Additionally, the expression of the nonsecreted protein CK18 and the representative hepatocyte marker albumin was upregulated in the constructs printed with GelXA LAMININK521 (Supplementary Figure S1E) compared to the other constructs using different bioink types. This result suggested that the encapsulated cells not only proliferated but also expressed constant hepatic markers in the bioprinted hepatic constructs over 14 days (Figure 1G). These findings highlight that GelXA LAMININK521 is the most appropriate scaffold for HepG2 cells to establish 3D-printed hepatic constructs resembling hexagonal liver lobules with improved hepatic properties and sufficient matrix components.

### 3.2. Spinning Culture Conditions Enable a Long-Term Culture Period of Bioprinted Hepatic Constructs with Consistent Hepatic Expression and Functionality of Encapsulated Cells

**Figure 2:**
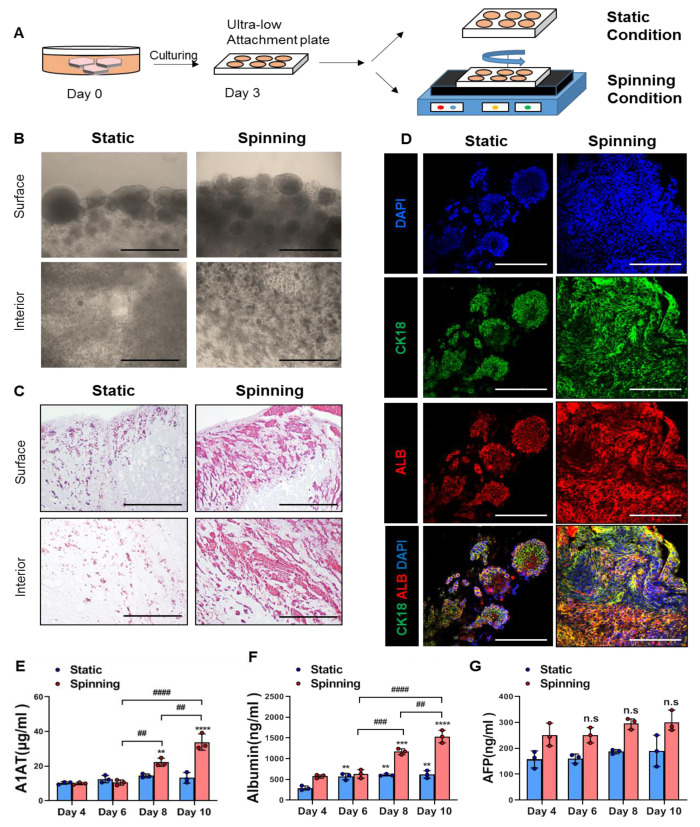
Encapsulated cells in bioprinted hepatic constructs revealed increased proliferative potential and further compacted liver parenchyma under spinning compared to static conditions. (**A**) Schematic diagram of bioprinted hepatic constructs. Rotatory culture condition generated by orbital shaker was designated as “spinning”, whereas the condition without rotating was referred to as “static”. Rotation was performed at 60 rpm. (**B**) Morphology of encapsulated cells located at the surface and interior areas of bioprinted hepatic constructs on day 14. Scale bar = 500 μm. (**C**) Representative H&E staining images showing the localization of encapsulated cells at the surface and interior areas of bioprinted hepatic constructs under static and spinning conditions on day 14. Scale bar = 500 μm. (**D**) Representative immunofluorescence images of bioprinted hepatic constructs. Sections were stained with cytokeratin 18 (green) and albumin (red) antibodies. Hepatic expression of HepG2 cells within bioprinted hepatic constructs was visualized on day 14 of culturing under static and spinning conditions. Scale bar = 250 μm. (**E**–**G**) ELISA for the secretion level of (**E**) human alpha-1 antitrypsin, (**F**) human albumin and (**G**) human alpha-fetoprotein in bioprinted hepatic constructs at indicated time points. The level was calculated every two days from days 4 to 10. Error bars represent the means ± S.D. from three separate experiments. One-way ANOVA followed by Bonferroni’s test was used for the statistical analysis. ** *p* < 0.01, *** *p* < 0.001 and **** *p* < 0.0001 show significant difference between day 4 and another day under the described culture condition. ## *p* < 0.01, ### *p* < 0.001 and #### *p* < 0.0001 indicate significance difference among each day under spinning condition. n.s: no significance.

Mechanical stimuli that are absent in static culture conditions contribute to the formation of key physiological structures and affect the growth and functionality of cells encapsulated in bioprinted structures [13]. Based on these facts, we adjusted the bioreactor-like system to recapitulate a microfluidic environment containing a mechanical stimulus mimicking blood flow and diffusion by circular shaking motion using an orbital shaker. HepG2 cells were encapsulated in bioprinted hepatic constructs and cultured under either static or spinning conditions where continuous media flow was induced using a platform orbital shaker at 60 rpm (Figure 2A). On day 14, bright-field images exhibited large spheroids, which were observed on the edge of bioprinted hepatic constructs, and spinning conditions contributed to the formation of larger amounts of spheroids (Figure 2B). In addition, cells located in the interior area of the bioprinted hepatic construct under spinning conditions showed more obvious aggregation and proliferation than those under static conditions (Figure 2B). Representative H&E images showed not only a large number of encapsulated cells but also spreading out of the whole bioprinted hepatic construct under spinning conditions compared to static conditions (Figure 2C). Next, we examined whether HepG2 cells in bioprinted hepatic constructs under spinning conditions could maintain hepatic expression and functionality for 14 days. The hepatic expression of HepG2 cells within bioprinted hepatic constructs was visualized with cytokeratin 18 and albumin antibodies. Compared to static conditions, spinning conditions contribute to the formation of larger amounts of CK18^+^ ALB^+^ cells in bioprinted hepatic constructs (Figure 2D). This result indicated that although bioprinting was performed using equal cell concentrations, the amounts of the cells remaining in the bioprinted hepatic constructs could differ depending on the culture conditions. Consistent with many observed morphological changes, there are also functional reinforcements in hepatic constructs cultured under 3D spinning conditions. In contrast to the increased secretion of human albumin (ALB) and alpha-1-antitrypsin (A1AT), the secreted level of alpha-fetoprotein (AFP) in the culture supernatant of bioprinted hepatic constructs did not show significant differences between static and spinning conditions from day 4 to day 10 (Figure 2E–G). These results are also consistent with the impact of spinning conditions in terms of the growth and maturity of encapsulated cells. Therefore, our results imply that spinning conditions enable HepG2 cells to grow with a consistent hepatic phenotype in bioprinted hepatic constructs over 2 weeks after bioprinting. Additionally, further studies on the secreted level of immature AFP protein compared to other proteins need to be conducted to more accurately explore the effects of spinning conditions on functional maturity utilized by different sources of human hepatocytes. Taken together, reinforcement of the clonogenic growth potential of encapsulated cells and functional maturation can be enhanced under spinning conditions. Additionally, the application of the orbital shaker provides continuous stimuli with appropriate diffusion of oxygen, nutrients and mechanical stimulation that supports defined and well-distributed encapsulated cells.

### 3.3. 3D Bioprinted Hepatic Constructs Show Efficient Changes in Cellular Characteristics under Spinning Conditions

**Figure 3:**
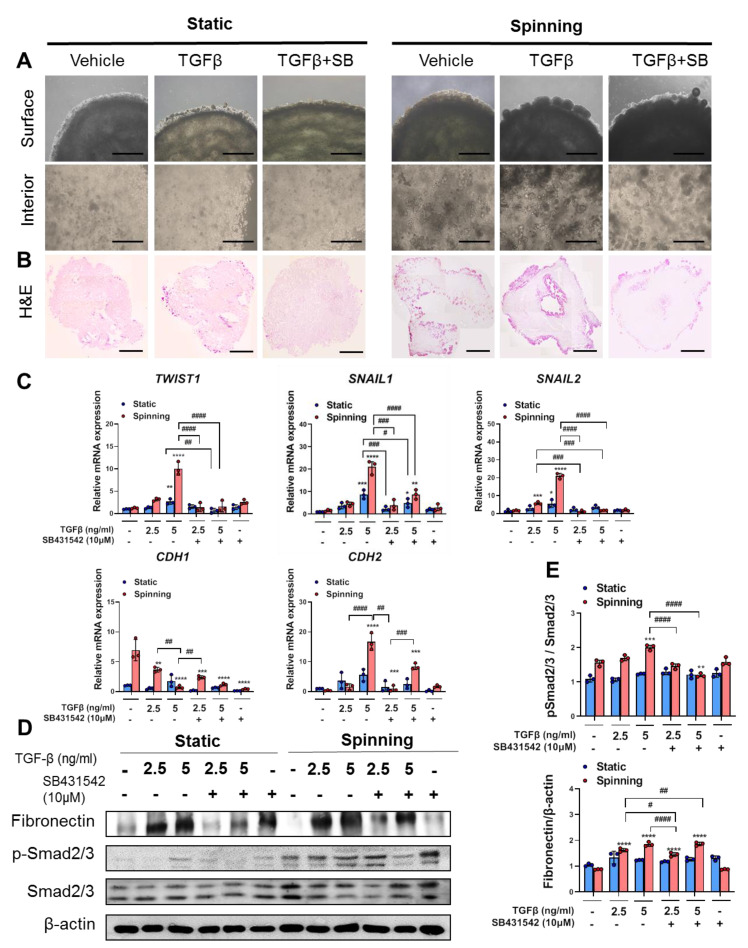
Spinning condition compared to static condition enhances TGF-β-induced epithelial-to-mesenchymal transition (EMT) pathway upregulation and inhibition by SB431542 treatment in bioprinted hepatic constructs. (**A**) Morphology of the surface and interior areas of bioprinted hepatic constructs in each group on day 10. Subgroups were divided into nontreated vehicle control, TGFβ-treated and combination of TGFβ with SB431542. Scale bar = 1000 μm. (**B**) Representative view of growth based on images for H&E staining of bioprinted hepatic constructs treated with TGF-β or TGF-β and SB431542 together under spinning and static conditions on day 10. Scale bar = 1000 μm. (**C**) qRT-PCR analysis of *TWIST1*, *SNAIL1*, *SNAIL2*, *CDH1* and *CDH2* genes that are involved in the EMT pathway in bioprinted hepatic constructs under static and spinning conditions on day 10. Error bars represent the means ± S.D. from three separate experiments. One-way ANOVA followed by Bonferroni’s test was used for statistical analysis. * *p* < 0.05, ** *p* < 0.01, *** *p* < 0.001 and **** *p* < 0.0001 show significance between control and other group under indicated culture condition. # *p* < 0.05, ## *p* < 0.01, ### *p* < 0.001 and #### *p* < 0.0001 denote significance among each group under indicated culture condition. (**D**) Western blot analyses of total cell lysates from bioprinted hepatic constructs using anti-fibronectin, anti-phosphosmad2/3, anti-Smad2/3 and anti-β-actin antibodies. β-Actin served as a loading control. (**E**) Quantification of fibronectin expression normalized to β-actin and phoshpho-Smad2/3 expression normalized to Smad2/3. One-way ANOVA followed by Bonferroni’s test was used for statistical analysis. Error bars represent the means ± S.D. from three separate experiments. ** *p* < 0.01, *** *p* < 0.001 and **** *p* < 0.0001 indicate significance between control and other group under indicated culture condition. # *p* < 0.05, ## *p* < 0.01 and #### *p* < 0.0001 show significance among each group under indicated culture condition.

The epithelial-to-mesenchymal transition (EMT) process could contribute to hepatic fibrogenesis not only in chronic liver diseases, as reported in other organs, but also in acute liver disease [27,28]. Additionally, rapid migration of a large number of fibrogenic cells by EMT could be one of the phenomena by which liver injury occurs [12]. Before we started to establish an APAP-induced liver injury model, constructs were treated with TGFβ to induce EMT as reported previously [29]. To investigate whether spinning conditions enhance the EMT process in bioprinted hepatic constructs, at day 3 after bioprinting, constructs were treated either with TGFβ or with TGFβ in combination with its inhibitor SB431542 for 7 days. On the third day of TGFβ treatment (total culture day 6), morphological changes started to appear differently depending on location in both of the culture conditions. As a result of TGFβ treatment, the encapsulated cells in 3D bioprinted hepatic constructs migrated to the surface area and began to form larger spheroids compared to static conditions. However, cells in the interior area showed a slightly elongated cell morphology exclusively under spinning conditions. Compared with spinning conditions, obvious morphological differences after TGFβ treatment under static conditions were not observed (Figure 3A). H&E staining images for the whole bioprinted hepatic construct demonstrated distinct cell numbers and distributions among the areas followed by different culture conditions (Figure 3B). For quantitative real-time PCR analysis and Western blotting, treatment with different TGFβ concentrations (2.5 and 5 ng/mL) was performed to ensure that TGFβ concentration dose-dependently induced the EMT response in the bioprinted hepatic construct. In particular, under spinning conditions, different TGFβ concentrations confirmed dose-dependent EMT induction, as evidenced by gene expression analysis (Figure 3C). In contrast to static conditions, 2.5 ng/mL TGFβ treatment under spinning conditions had a significant effect on effectors related to EMT gene levels (Figure 3C). No significant differences in relative EMT-related gene levels were noted with different culture conditions in the control groups (Figure 3C). Furthermore, treatment with TGFβ and SB431452 simultaneously showed significant reversible levels of EMT-related gene expression under spinning conditions (Figure 3C). Western blotting of fibronectin and phospho-smad2/3 demonstrated that bioprinted hepatic constructs cultivated under spinning conditions were more dose-dependently responsive to TGFβ signaling than the control groups (Figure 3D,E). Based on these results, spinning conditions accelerate exogenic chemically induced microenvironmental signaling. Therefore, these bioprinted hepatic constructs cultivated under spinning conditions allow us to predict possible drug effects to understand the underlying action mechanism and provide the potential possibility for establishing a drug-induced liver injury model.

### 3.4. The Dynamic Microenvironment Generated by an Orbital Shaker Enables Artificial Liver Construction as a Liver Toxicity Test Model

**Figure 4:**
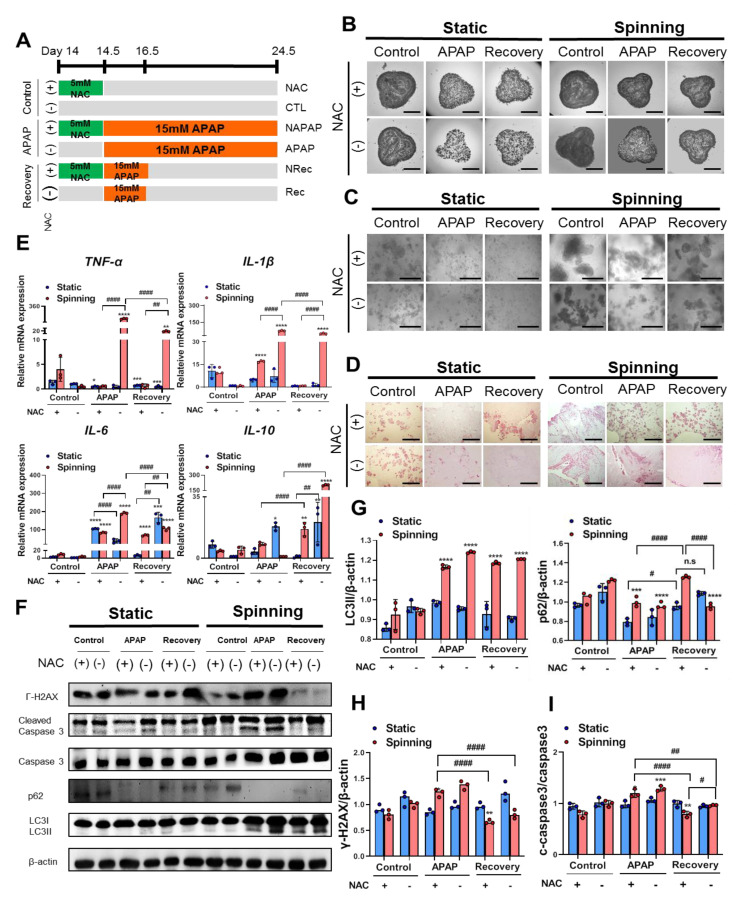
Spinning condition increased APAP-induced hepatotoxicity and prevention by administration of N-acetylcysteine (NAC) in bioprinted hepatic constructs via apoptosis and autophagy signaling. (**A**) Schematic diagram illustrating the strategy for treatment with APAP and NAC in bioprinted hepatic constructs at the indicated time point. Groups were divided into control (without APAP for 8 days), APAP (with APAP treatment for 8 day) and recovery (with APAP treatment for 2 days and without APAP treatment after the 2nd day). Control, APAP and recovery groups were subdivided with administration of NAC for 12 h before APAP treatment in control (NAC), APAP (NAPAP) and recovery (NRec) under static or spinning conditions. (**B**,**C**) Representative bright-field images represent (**B**) growth of bioprinted hepatic constructs and (**C**) magnified self-generated spheroids in bioprinted hepatic constructs on day 24.5. (**B**) Scale bar = 2500 μm. (**C**) Scale bar = 500 μm. (**D**) Magnified representative H&E images of bioprinted hepatic constructs on day 24.5 after acetaminophen and NAC treatment. Scale bar = 1000 μm. (**E**) qRT-PCR validation of *TNF-α*, *IL-1β*, *IL-6* and *IL-10* inflammatory response-related genes followed by APAP and NAC treatment on day 24.5. Error bars represent the means ± S.D. from three separate experiments. One-way ANOVA followed by Bonferroni’s test was used for the statistical analysis. * *p* < 0.05, ** *p* < 0.01, *** *p* < 0.001 and **** *p* < 0.0001 represent significance between CTL and other group under indicated culture condition. ## *p* < 0.01 and #### *p* < 0.0001 show significance among each group under indicated culture condition. (**F**) Western blot analyses of total cell lysates from bioprinted hepatic constructs treated with NAC and APAP using anti-phospho-histone H2A.X (Ser139), anti-cleaved caspase-3, anti-caspase-3, anit-p62, anti-LC3II, anti-LC3III and anti-β-actin antibodies. β-Actin served as a loading control. (**G**–**I**) (**G**) Quantification of p62, LC3II and (**H**) pH2AX expression levels normalized to β-actin and (**I**) cleaved-caspase-3 expression level normalized to caspase-3. Error bars represent the means ± S.D. from three separate experiments. One-way ANOVA followed by Bonferroni’s test was used for statistical analysis. ** *p* < 0.01, *** *p* < 0.001 and **** *p* < 0.0001 show significance between CTL and other group under indicated culture condition, and # *p* < 0.05, ## *p* < 0.01 and #### *p* < 0.0001 indicate significance among each group under indicated culture condition.

Because we observed that reinforcement of TGFβ induced the EMT process under spinning conditions, we hypothesized that drug responses in bioprinted hepatic constructs could also be improved depending on culture conditions. To ensure that the drug effect was in accordance with culture conditions, two drugs were selected: acetaminophen (APAP) for the induction of hepatotoxicity and N-acetylcysteine (NAC) for the prevention of APAP-induced hepatotoxicity. The confluency of encapsulated cells and the number of spheroids in the bioprinted hepatic constructs affect drug metabolism [19]. Hence, we introduced spinning conditions for 14 days to generate a large number of spheroids and provided similar confluency of encapsulated cells in all the bioprinted hepatic constructs. Extended treatment with NAC hinders hepatic function and impairs recovery potential from APAP-induced hepatotoxicity [30]. Thus, we treated bioprinted hepatic constructs with NAC 12 h before APAP treatment at the indicated time point (Figure 4A). Under spinning conditions, the control groups showed that a larger number of larger spheroids remained inside the bioprinted hepatic constructs (Figure 4B). However, following APAP treatment for 2 days, we observed reduced confluency of small spheroids in whole bioprinted control groups regardless of culture conditions. After APAP treatment, we divided the group into continuous APAP treatment (NAPAP) and no APAP treatment (Figure 4C). However, significant morphological changes under static conditions compared with NRec and Rec as well as NAPAP and APAP resulting from administration of NAC were not observed (Figure 4B). Representative H&E images show damaged cell nuclei in the APAP group, especially under spinning conditions. APAP overdose induces severe liver injury accompanied by reactive metabolite formation, mitochondrial damage, cell death via apoptosis and autophagy signaling [31,32]. To assess the effect of APAP and NAC treatment depending on culture conditions, Western blot analysis was performed (Figure 4D). As expected, RT-PCR and Western blot analysis of bioprinted hepatic constructs subjected to drug treatment under spinning conditions showed higher levels of the proinflammatory cytokines TNF-α, IL-1β and IL-6 in the APAP and Rec groups compared with the static group [31,33]. On the other hand, IL-10 expression was enhanced in Rec under spinning conditions (Figure 4E). In addition, bioprinted hepatic constructs showed increased apoptosis levels, as observed by the expression of cleaved caspase-3 with constant APAP treatment (Figure 4F,I). Additionally, increased autophagy levels were identified based on a reduction in p62 and an increase in LC3II in the APAP group under spinning conditions (Figure 4F,G). In contrast to static culture conditions, apoptosis and autophagy effects were reinforced in NAPAP compared to APAP. In particular, the effect of NAC administration was significant in the NRec and Rec groups. Consistent with the activation of autophagy and apoptosis pathways with persistent APAP treatment, bioprinted hepatic constructs under spinning conditions showed enhanced levels of gamma H2AX, a hallmark of DNA double-strand breaks (Figure 4F,H). Furthermore, administration of NAC to bioprinted hepatic constructs prevented APAP-induced hepatotoxicity via apoptosis and autophagy signaling. In particular, the NRec and Rec groups exhibited regenerating liver phenomena confirmed by dephosphorylated H2AX expression from acetaminophen-induced hepatotoxicity [34]. Based on these results, spinning conditions contribute to efficient drug sensitivity ranging from APAP-induced hepatotoxicity to prevention of hepatotoxicity by administration of NAC compared to static conditions.

### 3.5. Functional Evaluation of Bioprinted Hepatic Constructs Revealed That Hepatotoxicity Induced by APAP and Prevented by NAC Was Enhanced under Spinning Conditions

**Figure 5:**
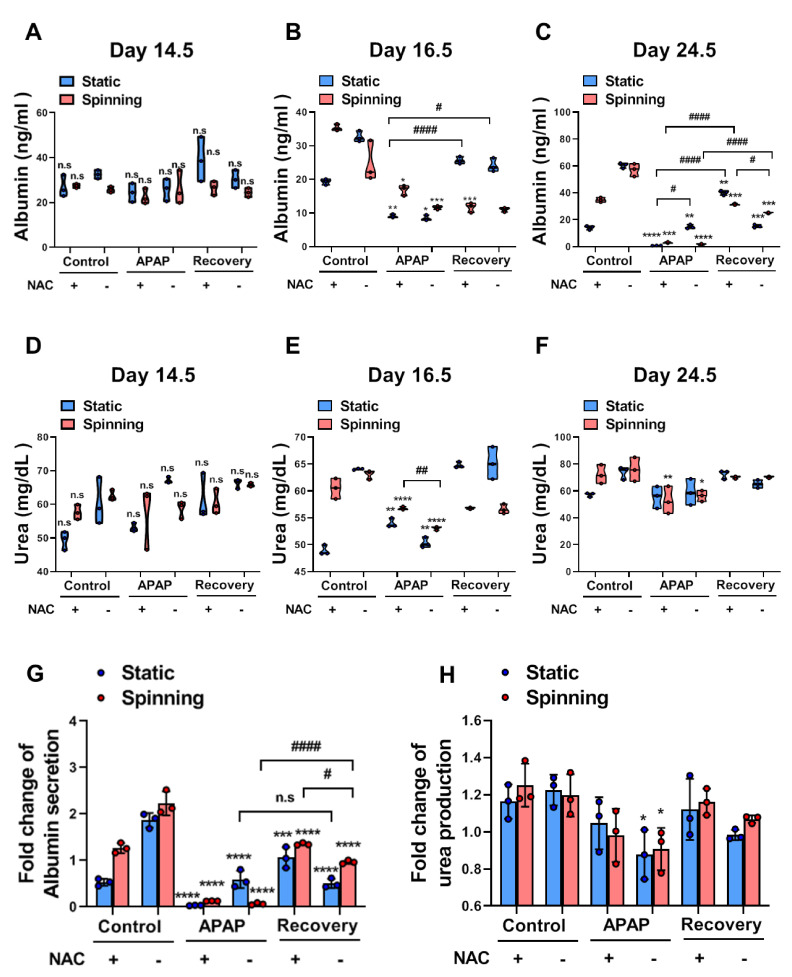
Functional evaluation for APAP-induced hepatotoxicity and prevention by NAC administration showed enhancement of drug sensitivity in the bioprinted hepatic constructs under spinning compared to static conditions. (**A**–**C**) ELISA for albumin secretion levels in bioprinted hepatic constructs in each indicated group cultured under static and spinning conditions on (**A**) day 14.5, (**B**) day 16.5 and (**C**) day 24.5. Culture supernatants were collected every other day starting on day 1 after bioprinting except during the NAC treatment period. Error bars represent the means ± S.D. from three separate experiments. One-way ANOVA followed by Bonferroni’s test was used for statistical analysis. * *p* < 0.05, ** *p* < 0.01, *** *p* < 0.001 and **** *p* < 0.0001 indicate significance between CTL and other group under indicated culture condition. # *p* < 0.05 and #### *p* < 0.0001 show significance among each group under indicated culture condition. (**D**–**F**) Urea production levels in bioprinted hepatic constructs in each indicated group cultured under static and spinning conditions on (**D**) day 14.5, (**E**) day 16.5 and (**F**) day 24.5. * *p* < 0.05, ** *p* < 0.01 and **** *p* < 0.0001 indicate significance between CTL and other group under indicated culture condition. ## p < 0.01 shows significance among each group under indicated culture condition. (**G**,**H**) (**G**) Fold change in albumin secretion and (**H**) urea production in culture supernatant of bioprinted hepatic constructs in each group on day 24.5 normalized to day 14.5 under static and spinning conditions. * *p* < 0.05, *** *p* < 0.001 and **** *p* < 0.0001 show significance between control and other for each group under indicated culture condition. # *p* < 0.05 and #### *p* < 0.0001 indicate significance among each group under indicated condition.

To examine whether spinning conditions improve the functionality of bioprinted hepatic constructs for drug sensitivity, functional evaluation of bioprinted hepatic constructs was performed by assessing the level of albumin secretion and urea synthesis. The liver synthesizes albumin, which plays a role in carrying hormones, vitamins and enzymes to maintain homeostasis in the body and is secreted into the blood. Additionally, the liver is the primary site for the urea cycle, which converts highly toxic ammonia to urea for excretion. Therefore, the levels of albumin secretion and urea synthesis in blood or culture supernatant represent liver functionality. Specifically, low levels of albumin and urea indicate liver dysfunction. Under spinning conditions, control groups showed not only increased levels of secreted albumin but also increased urea production in culture supernatants compared to the static group. Additionally, APAP treatment in bioprinted hepatic constructs significantly decreased the level of secreted albumin and urea production (Figure 5A–F). Due to inaccuracies during extrusion-based bioprinting, unequal amounts of cells could lead to a misunderstanding of drug effects following different culture conditions. Thus, the fold change in albumin secretion in all groups was examined on day 10 and normalized to the secreted level in the culture supernatant after NAC treatment (Figure 5G,H). Both the fold change of urea production and albumin secretion indicated a more accurate drug effect under spinning conditions because no significant differences were observed in all groups on day 0.5. On day 16.5, the APAP and Rec groups were at a similar stage of acetaminophen-induced hepatotoxicity; however, bioprinted hepatic constructs cultivated under static conditions showed inconsistent changes in the level of albumin secretion and urea synthesis (Figure 5B,E). Taken together, these results suggest that spinning conditions increased the susceptibility of drugs in bioprinted hepatic constructs immediately and clearly.

## 4. Discussion

In vitro hepatic models are used by researchers to accurately predict and assess drug response and screening. It is important to represent the high metabolism of drugs and toxins resulting from the complex liver microenvironment [13]. Unfortunately, in most of the currently developed in vitro hepatic models supported by biocompatible scaffolds, it is difficult to evaluate precise drug effects due to the slow diffusion of nutrients and oxygen between media and encapsulated cells. To address the limitations of current 3D cell culture models for hepatotoxicity evaluation [7,8], we developed a 3D bioprinted hepatic model incorporated with a dynamic microenvironment that enables the accurate evaluation of hepatotoxicity drug responses.

To achieve successful bioprinting and develop a decent predictive model, the choice of an appropriate scaffold is an important factor to be considered. Recently, applied polymeric hydrogels have revealed poor stability and low printing accuracy; therefore, various biomixtures are being developed to enhance pre- and postprinting features as well as cytocompatibility and after-printing cellular development [1]. In this study, GelXA bioinks, which constitute a gelatin methacrylate (GelMA)-based biocompatible mixture in combination with xanthan gum and alginate, were used to enhance printability [35]. Given that GelMA has remarkable potential in controlling temporal and spatial properties, it is widely used as a 3D scaffold that plays a critical role in cell adhesion, biocompatibility and biodegradability [36]. The addition of different types of laminin proteins to GelXA bioink needs to be optimized followed by the cellular properties of encapsulated cells. Laminins promote cell adhesion in the regenerating liver and provide the liver stem cell niche [24,37]. Additionally, laminin-511- and laminin-521-based matrices support efficient hepatic specification of human pluripotent stem cells and promote the acquisition of hepatic functions [38]. In our study, among different commercially available types of bioinks based on gelatin methacrylate, xanthan gum, alginate and laminin, GelXA LAMININK521 was considered the most appropriate. It induced clonogenic expansion and provided spatial organization of cells with enhancement of hepatic phenotypes in bioprinted hepatic constructs. Cells grown in our 3D models printed with GelXA LAMININK521 formed spheroids with strong cell–cell and cell–ECM interactions within the extracellular matrix. Spheroids containing proliferating, quiescent, hypoxic and necrotic cells more closely mimic the in vivo microenvironment due to the different cell development stages instead of their existence in the same stage of the cell cycle [39]. Consequently, it seems to exhibit an ineffective drug response in contrast to 2D models that are equally exposed to nutrients and drugs in growth media. Given that the 3D model depends on simple diffusion due to the absence of blood vessels, limited penetrability of nutrients and drugs in the media is noted. As a result, restricted oxygenation and nutrition environments in bioprinted hepatic constructs cause hypoxic conditions, which influence local pH and further hamper robust drug responses [39,40]. These limitations were not sufficient to overcome bioink support and led to the development of another strategy in our model.

The major problem that we aimed to overcome in this study is the lack of sufficient supplementation of nutrients and oxygen, which disturbs accurate drug metabolism. We introduced spinning conditions through the use of a rotatory platform shaker to 3D bioprinted hepatic constructs to exchange nutrients and oxygen efficiently and further extend the culture period. In our study, the HepG2 cell line, which has high proliferation capacity and consistent hepatic functionality, was chosen for encapsulation in bioink to represent the hepatocyte population in the parenchyma and achieve metabolic activity similar to that of the liver. Despite incompatible functionality compared to primary hepatocytes, HepG2 has advantages in easy accessibility, a long-term stable phenotype and strong survival rates during the bioprinting process. As reported previously, prolonged cultivation time of HepG2 cells rather than 3D culture contributed to increased drug metabolism [41]. As a result, bioprinted hepatic constructs cultivated under spinning conditions enable close representation of the in vivo state with reinforcement of clonogenic growth potential and functionality assessed by hepatic marker profiling and protein secretion levels.

Prior to modeling APAP-induced liver injury, we tested whether spinning conditions progress through the TGFβ-induced EMT pathway and whether this process is reversed by SB431542 treatment. Bioprinted hepatic constructs cultivated under spinning conditions showed breakdown of cell–cell interactions confirmed by TGFβ-induced EMT and reinforcement of drug sensitivity for hepatotoxicity based on gene-level analysis and protein expression. This finding might be explained by mitigation of hypoxic conditions followed by rotation, which leads to reinforcement of chemically induced signals and drug-induced metabolism. Consequently, under spinning conditions, prolonged APAP treatment leads to enhanced hepatotoxicity by modulating the inflammatory response as well as autophagy and apoptosis signaling and further causes necrosis in the encapsulated cells compared to static cells. In contrast, 3D bioprinted hepatic constructs with NAC under static conditions did not show a significant relative decline in apoptosis levels in contrast to spinning conditions, which is similar to that noted in a previous study [42]. This finding indicates that under static conditions, bioprinted hepatic constructs show large spheroids that are less susceptible and are not subjected to apoptosis when exposed to drugs [43]. Additionally, an extended culture period resulted in grape-like morphological changes that were responsible for poor cell–cell adhesion [39]. Although short-term APAP-induced hepatotoxicity was not protected by administration of NAC regardless of culture conditions, spinning conditions enhanced the consistent APAP-induced hepatotoxicity effect in contrast to static conditions. Several studies evaluated continuous rotating effects mainly using perfusion to establish a more physiologically relevant in vitro 3D hepatic model [20,44]. However, the major limitations of these systems include difficulties in standardizing and scaling up technology. Additionally, these systems require additional micropumps, tubes and valves to deliver drugs into the targeted area, which complicates the process of establishment [45].

Despite the encouraging application of our model for drug toxicity assessments, many factors should be considered in this study, i.e., restricted cell sources and the absence of exquisite design for representing liver anatomy in in vitro bioprinted hepatic constructs. Nevertheless, in particular, we recapitulated liver injury and repair phenomena, increasing susceptibility to acetaminophen toxicity and prevention by administration of N-acetylcysteine (NAC) in bioprinted hepatic constructs. It is difficult to represent complex liver regeneration in vivo. In liver injury, hepatic regeneration responds to inflammatory cytokines, such as TNFα and IL-6 produced by Kupffer cells, to reconstitute the liver by rapidly inducing cells to enter the cell cycle [46,47]. Additionally, the absence of liver stem/progenitor cells, which contribute to liver regeneration, reveals the need for reinforcement in further studies [48,49].

However, given that we encapsulated a single, representative cell type of liver in bioprinted hepatic constructs without sophisticated design, precise dynamic environmental effects can be evaluated in terms of maintaining the growth rate and functionality as well as metabolizing drugs. Based on these results, an ideal new generation in vitro model with multicellular organization can be established to parallel the main process responsible for pharmacokinetics and complete modeling of cell–cell interactions [50]. Indeed, the cascade of immune responses from multiple cell types to injury leads to the development of a successful alternative method that enables the replacement of animal models [51]. Further incorporation of the advanced development of robust human induced pluripotent stem cell (hiPSC) differentiation approaches is encouraging, and a patient-derived liver model can offer great potential for advancement of personalized disease modeling and medicine. This simple but efficient strategy will improve existing successful models for further in vitro research with simultaneous applications.

In conclusion, the successful combination of applied extrusion-based bioprinting technology with laminin-521-enriched biocompatible bioink facilitates the fabrication of a 3D in vitro model capable of supporting the structural integrity and tunability of mechanical properties in a precisely controlled manner. Bioengineered hepatic constructs with controlled stiffness respond weakly to chemically induced microenvironmental signals and impair metabolic processes under static conditions, but these processes are improved upon the incorporation of a dynamic environment generated by an orbital shaker. The dynamic environment incorporated into these bioprinted hepatic constructs promotes a long-term culture period, resulting in enhanced functionality. Indeed, the dynamic environment replicates the complex in vivo microenvironment, including physiological and mechanical cues, permitting the study of induced fibrogenesis mechanisms at the cellular level and evaluation of the effects of drugs. Additionally, under spinning conditions, the increased number of existing healthy spheroids that play an important role in drug metabolism and the remarkable viability of encapsulated cells suggest that this combined strategy in bioprinted hepatic constructs can be utilized for accurate drug evaluation for hepatotoxicity prevention and induction. Indeed, enhancement of drug susceptibility verified by APAP-induced hepatotoxicity and alleviation by NAC administration through spinning conditions allowed the development of a novel culture system to study the human liver in the dish. Furthermore, this unique culture system will provide an excellent opportunity to scale up the advantages of 3D bioprinting technology with enhanced functionality and proliferation capacity compared to currently existing in vitro hepatic models. The combined application of 3D printing technology and subsequent spinning conditions improves the quality of 3D modeled tissue, is not resource-intensive and reduces labor costs and the need for reagents for further commercialization of the method in research applications and personalized medicine or cell therapy.

## Data Availability

There are no data relevant to accession codes or unique identifiers that are not publicly available. All generated data are included in the manuscript and available upon reasonable request to K.-S.K.

## Supplementary Materials

The following are available online at https://www.mdpi.com/article/10.3390/cells10051268/s1, Figure S1. GelXA LAMININK521 is the most appropriate bioink for encapsulated cells to maintain inherent phenotype within bioprinted hepatic constructs; Figure S2. Characterization of self-generated spheroids from 3D bioprinted hepatic constructs; Figure S3. Whole Western blots that show all bands with markers for fibronectin, pSmad2/3, Smad2/3 and β-actin; Figure S4. Whole Western blots that show all bands with markers for γH2AX, cleaved caspase 3, caspase 3, p62, LC3β and β-actin; Table S1. List of primary antibodies; Table S2. List of oligonucleotide primers used in real-time PCR experiments.
