# Supplementary material for: Establishing a 3D In Vitro Hepatic Model Mimicking Physiologically Relevant to In Vivo State
Source: Cells. 2021 May 20;10(5):1268. doi: 10.3390/cells10051268 (PMC8161177; doi:10.3390/cells10051268)
Supplement: Supplementary file 1 [file cells-10-01268-s001.zip › cells-1204622-supplementary.pdf]

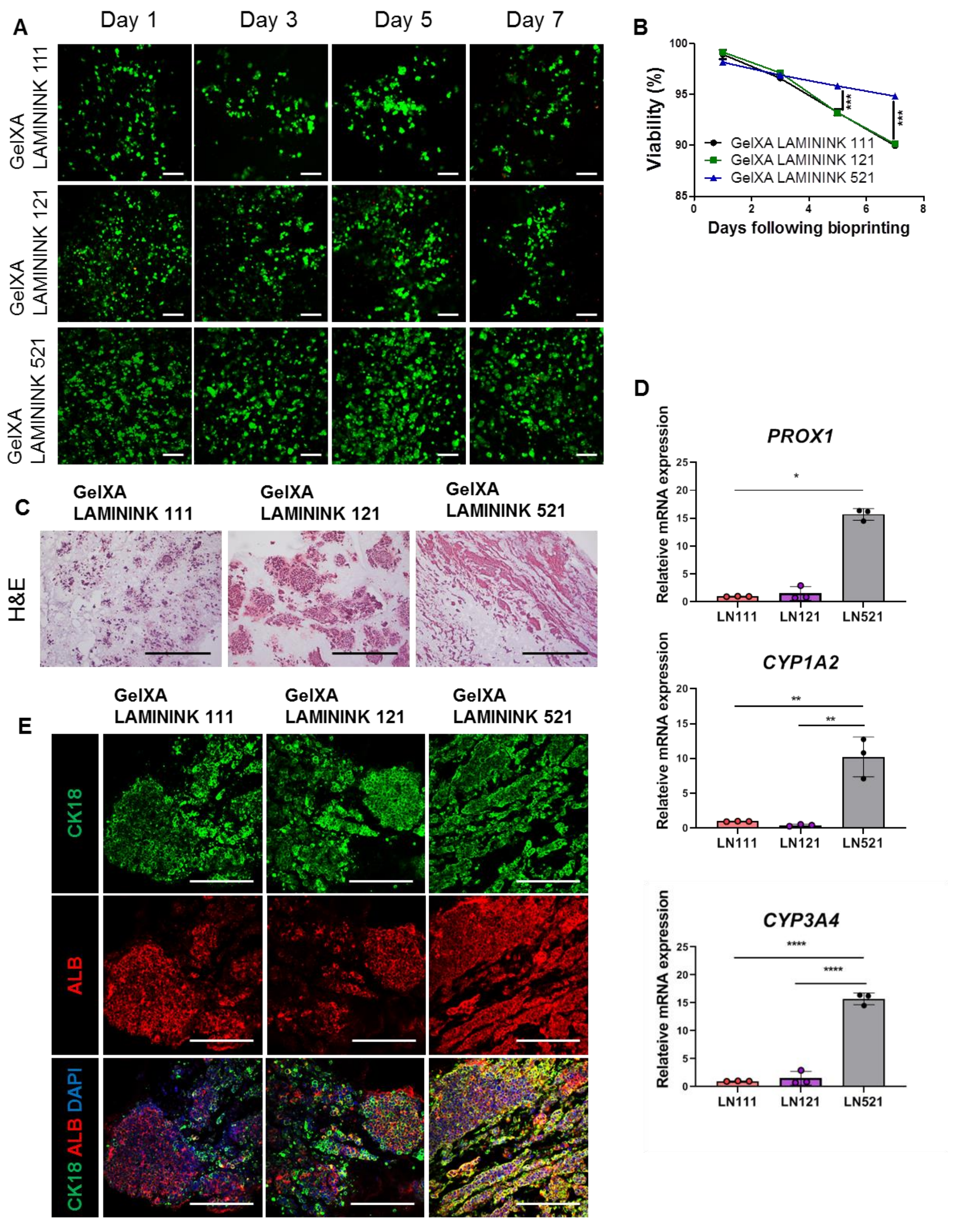

Supplementary figure 1. GelXA LAMININK521 is the most appropriate bioink for encapsulated cells to maintain inherent phenotype within bio-printed hepatic constructs.

(A) Representative images for live/dead staining with calcein AM (green) and PI (red) in bio-printed hepatic constructs generated using GelXA LAMININK111, GelXA LAMININK121 and GelXA LAMININK521 on days 1, 3, 5 and 7. Scale bar = 200 μm.

(B) Quantification of encapsulated cells viability in bio-printed hepatic constructs generated using GelXA LAMININK111, GelXA LAMININK121 and GelXA LAMININK521. Line graph represents viability of the cells in different types of bioink for 7 days. Greater than 95% viability was noted in all groups, and no significant differences were noted between one day after bioprinting. On day 7, cell viability was the highest in the bio-printed hepatic construct printed with GelXA LAMININK521.

(C) Representative H&E images for bio-printed hepatic constructs generated using GelXA LAMININK111, GelXA LAMININK121 and GelXA LAMININK521 on day 14. Scale bar = 250 μm.

(D) Gene expression profiles showing *PROX 1*, *CYP1A2*, and *CYP3A4* expression levels in bio-printed hepatic constructs generated using GelXA LAMININK111, GelXA LAMNINK121 and GelXA LAMININK521. All error bars represent the means ±S.D. from three separate experiments. One-way ANOVA followed by Bonferroni's test were used for the statistical analysis. *$p < 0.05$ and **$p < 0.01$.

(E) Representative immunofluorescence images of 3D bio-printed hepatic constructs generated in different bioinks. Sections were stained with CK18 (green) and ALB (red) on day 14. Scale bar=250 μm.

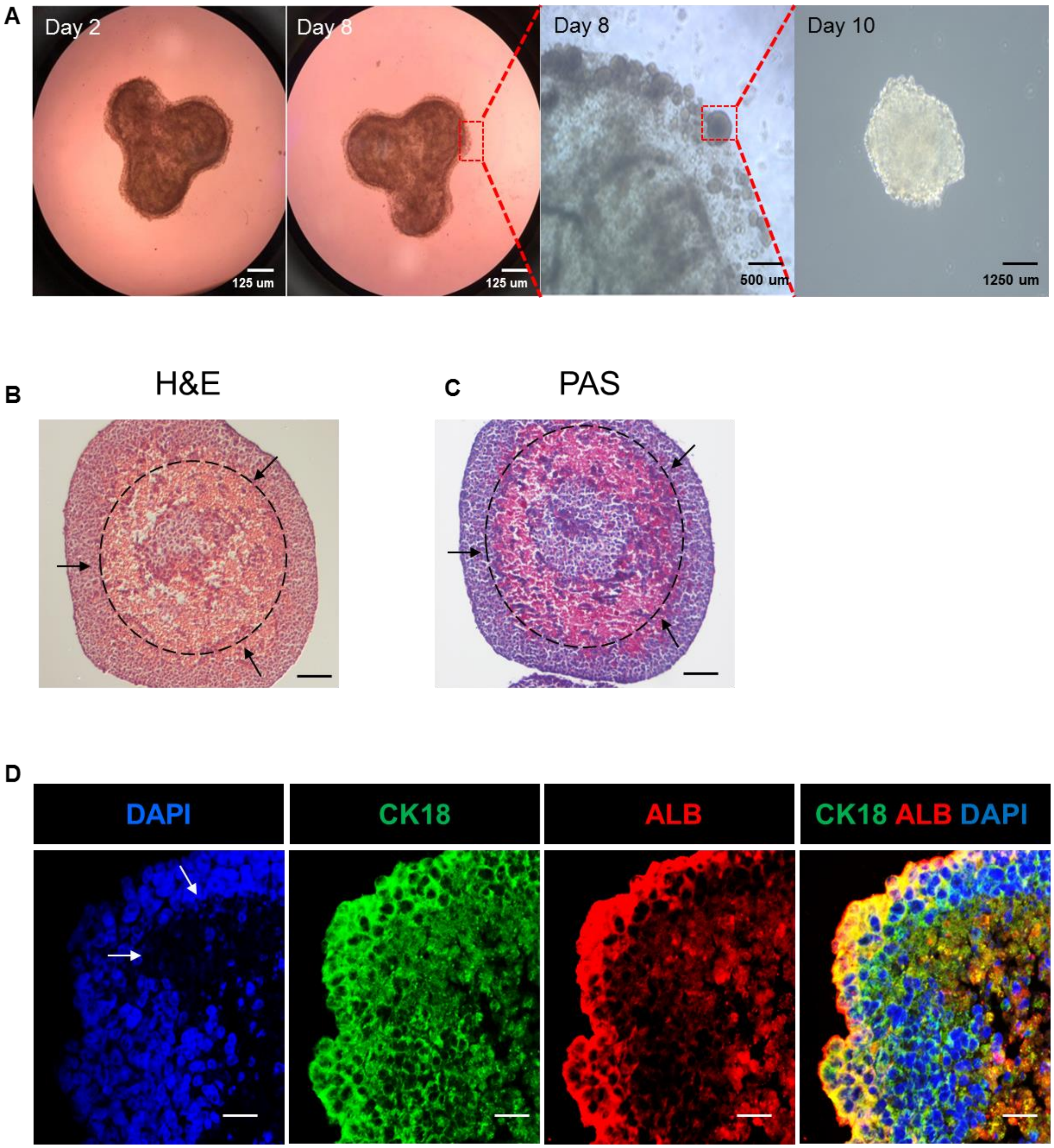

Supplementary figure 2. Characterization of self-generated spheroids from 3D bio-printed hepatic constructs.

(A) Representative growth based on bright field images for 3D bio-printed hepatic construct on days 2 and 8. Magnified bright field images show a spheroid generated from bio-printed hepatic

constructs that migrated from bio-printed structures on day 8 and cultured in 96 well ultra low-attachment plates for 7 days (total culture day 15).

(B-C) Representative images of spheroids from bio-printed hepatic constructs stained with (B) H&E and (C) Periodic acid schiff (PAS) on total culture day 15. Scale bar = 100 μm. Arrow heads indicate the necrotic cell debris and damaged cells.

(D) Representative immunofluorescence images of spheroids released from 3D bio-printed hepatic construct on total culture day 15. Sections were stained with ALB (red) and CK18 (green). Scale bar = 100 μm. Arrow heads indicate the necrotic cell debris and damaged cells.

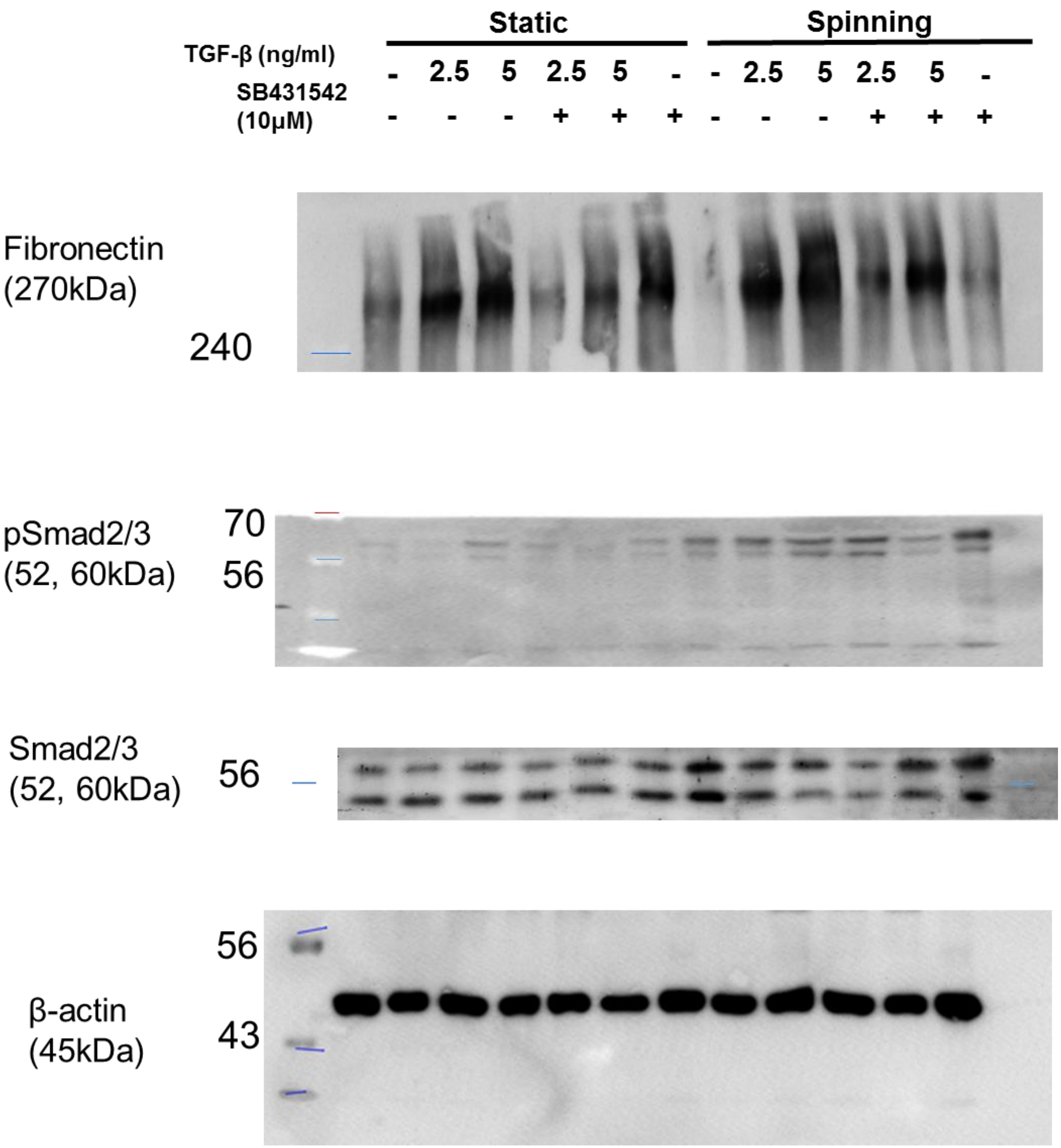

Supplementary figure 3. Whole western blots that show all bands with marker for Fibronectin, pSmad2/3, Smad2/3 and β-actin.

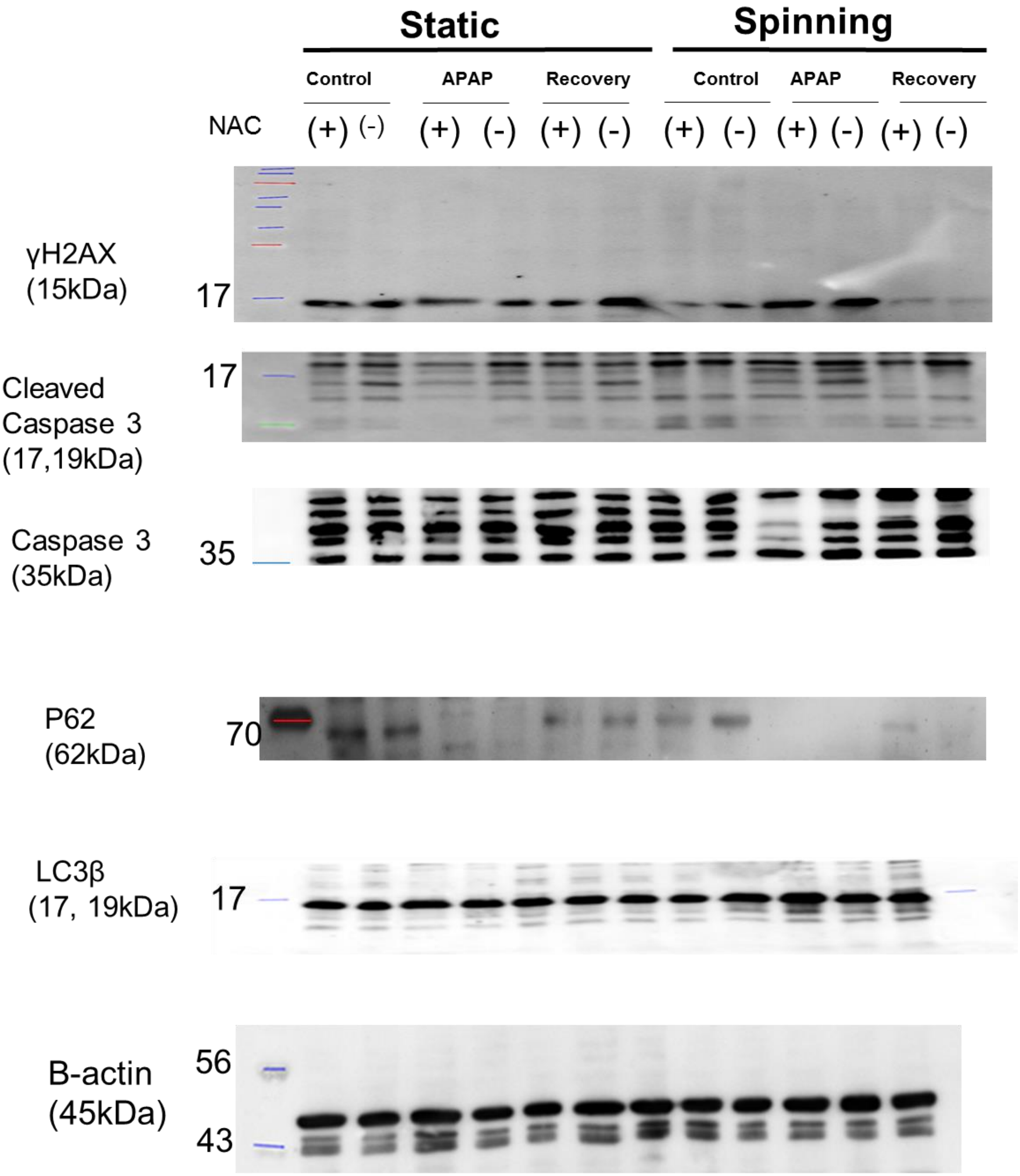

Supplementary figure 4. Whole western blots that show all bands with marker for γH2AX, Cleaved caspase 3, caspase 3, p62, LC3β and β-actin.

Table 1. List of primary antibodies

Description	Company	Product number
Cytokeratin 18	Millipore	MAB3234
Albumin	Genetex	102419
Cleaved caspase 3	Cell signaling	9664
Caspase-3	Cell signaling	9662s
LC3B	Novus	NB100-2331
p62	BD Bioscience	610832
Phospho Histone H2A.X	Merck	05-636
Fibronectin	Abcam	ab2413
phosphoSmad2/3	Cell signaling	8828
Smad2/3	Cell signaling	8685
GAPDH	Santa Cruz	sc-47724

Table 2. List of oligonucleotide primers used in real-time PCR experiments

Gene	Forward<br>Primer Sequence (5'-3')	Reverse<br>Primer Sequence (5'-3')
PROX1	CAGCCCGAAAAGAACAGAAG	GGGTCTAGCTCGCACATCTC
CYP1A2	GTCATTGGTGCCATGTGCTT	TTAGGCAGGTAGCGAAGGATG
CYP3A4	CAATAAGGCACCACCCACCT	CACCACCCCTTTGGGAATGA
TWIST1	TCTCAAGAGGTCGTGCCAATC	TGACTATGGTTTTGCAGGCCA
SNAIL1	TAGCGAGTGGTTCTTCTGCG	AGGGCTGCTGGAAGGTAAAC
SNAIL2	ATCACTGTGTGGACTACCGC	TCACTCGCCCCAAAGATGAG
CDH1	TGGACCGAGAGAGTTTCCCT	ACGACGTTAGCCTCGTTCTC
CDH2	AGGCGTTATGTGTGTATCTTCACT	CTGCCACTTGCCACTTTTCC
TNFα	CACAGTGAAGTGCTGGCAAC	GATCAAAGCTGTAGGCCCCA
IL-1β	CTGAGCTCGCCAGTGAAATG	TGTTTAGGGCCATCAGCTTCA
IL-6	TCAATATTAGAGTCTCAACCCCCA	TTCTCTTTCGTTCCCGGTGG
IL-10	GGCACCCAGTCTGAGAACAG	ACTCTGCTGAAGGCATCTCG
